# Monitoring Anthropogenic Ocean Sound from Shipping Using an Acoustic Sensor Network and a Compressive Sensing Approach †

**DOI:** 10.3390/s16030415

**Published:** 2016-03-22

**Authors:** Peter Harris, Rachel Philip, Stephen Robinson, Lian Wang

**Affiliations:** 1 National Physical Laboratory, Hampton Road, Teddington, Middlesex TW11 0LW, UK; stephen.robinson@npl.co.uk (S.R.); lian.wang@npl.co.uk (L.W.); 2 Mathematical Institute, University of Oxford, Andrew Wiles Building, Woodstock Road, Oxford OX2 6GG, UK; Rachel.Philip@maths.ox.ac.uk

**Keywords:** ocean sound, acoustic sensor network, compressive sensing, source reconstruction

## Abstract

Monitoring ocean acoustic noise has been the subject of considerable recent study, motivated by the desire to assess the impact of anthropogenic noise on marine life. A combination of measuring ocean sound using an acoustic sensor network and modelling sources of sound and sound propagation has been proposed as an approach to estimating the acoustic noise map within a region of interest. However, strategies for developing a monitoring network are not well established. In this paper, considerations for designing a network are investigated using a simulated scenario based on the measurement of sound from ships in a shipping lane. Using models for the sources of the sound and for sound propagation, a noise map is calculated and measurements of the noise map by a sensor network within the region of interest are simulated. A compressive sensing algorithm, which exploits the sparsity of the representation of the noise map in terms of the sources, is used to estimate the locations and levels of the sources and thence the entire noise map within the region of interest. It is shown that although the spatial resolution to which the sound sources can be identified is generally limited, estimates of aggregated measures of the noise map can be obtained that are more reliable compared with those provided by other approaches.

## 1. Introduction

There has recently been increasing concern over the potential effects of anthropogenic acoustic noise on marine life, and the influence this noise poses for the sustainability of key marine species, biodiversity, ecosystems and the overall health of our seas [1,2,3]. Such noise-generating activities include offshore oil and gas exploration, developments of offshore platforms and services, marine renewable energy developments, dredging and aggregate extraction, construction activities, naval sonars, explosive decommissioning of offshore developments, and disposal of munitions and ordnance. In addition, the rapid increase in commercial shipping traffic has the potential to increase the background level of sound in the ocean. The effect on marine fauna of high amplitude sources may include physiological effects (e.g., damage to hearing) or behavioural effects (e.g., flight response or displacement from habitats). An increase in background noise level may also have chronic effects (e.g., masking of biologically produced sound vital for communication and foraging) [1,2,3].

This concern has motivated increased monitoring of ocean acoustic noise, with a number of recent studies being reported. Examples include studies of low frequency sound in the Northeast Pacific Ocean, which demonstrate an increase over the last 60 years [4,5,6,7,8]. An increase has also been observed in low frequency levels (2 dB to 3 dB in the range 5 Hz to 115 Hz) in the Indian Ocean over the last decade [9,10]. More recently, observations in the Northeast Pacific Ocean show a constant level or slightly decreasing trend in low frequency noise [11], and observations from deep-water stations installed in the Atlantic, Pacific and Indian oceans could not clearly identify common trends between the stations [12,13], making it difficult to make generalised statements about global changes in ocean noise. In general, deep water observations of low frequency trends are influenced by noise from ice breaking, whales and noise from geophysical surveys, but noise from ship traffic is also an influence [9,12] and the growth in ship traffic is widely considered as a potentially significant influence factor in global ocean noise levels [14,15].

There have been no long-term studies of ocean noise in the shallow water of the continental shelf, and trends are therefore unknown. However, the trends in noise levels in these regions, and the spatial distribution of the noise, is increasingly subject to regulation. For example, the European Union’s Marine Strategy Framework Directive (2008/56/EC) requires member states to monitor ocean acoustic noise in order to demonstrate Good Environmental Status [16]. Here, acoustic noise is considered to be unwanted sound from anthropogenic sources, and in the Directive it is explicitly classed as a pollutant. This has led to interest in improved understanding of the noise radiated by the sources, and in noise maps, which describe the spatial variation of the acoustic noise levels within a region of interest [17].

There are different approaches to estimating the acoustic noise map. One is based on modelling alone. It uses knowledge of the sources of sound that are known to be present and makes use of models for the acoustic output of those sources and for sound propagation in the ocean. Another is based on measurement alone. It makes use of a network of acoustic sensors at a set of discrete locations within the region of interest to record values of the sound produced by all sources (whether their locations are known or not). Recently, however, there has been interest in an approach that uses a combination of modelling and measurement [17,18,19]. In this paper consideration is given to how such an approach might be implemented using a method based on compressive sensing. It is demonstrated that the estimation of the acoustic noise map can be improved by aggregating appropriately measured data provided by a sensor network with measurement models, and without the need to improve the available hardware such as the individual sensors used for ocean sound monitoring.

The problem of estimating the acoustic noise map is divided into two steps. In the first step, *source reconstruction*, the measured values provided by the acoustic sensors in the network together with partial information about the sound sources are used to provide estimates of the source locations and the source levels, which describe the acoustic output of the sources. This step is formulated as a linear system of equations whose solution estimates the source level at each possible location of a source within the region of interest. Since the number of possible source locations is very large, the system of equations is highly underdetermined. However, the number of actual sources is known to be small, and so the solution vector should be sparse. Thus, the problem is akin to one of compressive sensing, and a sparsity-inducing algorithm can be used to obtain a solution. The second step, *sound prediction*, uses the solution to the source reconstruction problem to provide estimates of the mean squared sound pressure, which is related to acoustic intensity, at any location within the region of interest. In terms of these estimates, aggregated measures of the sound field, such as the spatially averaged sound pressure level, can be calculated.

To illustrate the methodology, a set of numerical simulation experiments is proposed and used to compare the approach with those based on modelling alone and measurement alone. The experiments are also used to quantify the uncertainties associated with estimates of the spatially averaged sound pressure level, and to investigate the influence of the design of the network of sensors on the results obtained. It is shown that although the spatial resolution to which the source reconstruction problem can be solved is generally limited, reliable estimates of the spatially averaged sound pressure level can be obtained, and therefore the approach can provide support to an implementation of the Marine Strategy Framework Directive.

The scenario chosen for the numerical simulation experiments is that of a sound field generated by ships (the sources of sound) contained within a commercial shipping lane in shallow water with a network of acoustic sensors located outside but adjacent to the shipping lane. A frequency interval of 40 Hz to 200 Hz has been chosen because this is the interval over which ship traffic noise is a dominant factor in the measured ocean ambient noise spectra. The necessary inputs for the experiments in terms of source levels and source locations are in general available for this scenario: estimates of typical source levels for commercial ships may be obtained from data in the scientific literature ([20] and Section 2.2), and the locations of the sources may be chosen based on data for realistic ship traffic density that is available from the Automated Identification System (AIS). AIS is an automatic tracking system used for identifying and locating ships by electronically exchanging data with other nearby ships, AIS base stations and satellites. The system provides data for the location, heading and speed of commercial ships of gross tonnage of 300 or more and all passenger ships regardless of size. The availability of the above data enables the methodology described in this paper to be applied in principle to real-world scenarios. Note that although the work described is restricted to the ship traffic scenario, the method can in principle be extended to other sources for which data exists for their locations and source acoustic output.

Solving the source reconstruction problem is generally incidental to the problem of monitoring anthropogenic ocean sound. However, there are applications, for example, concerned with the use of a passive network of acoustic sensors to identify and track sound sources, for which it is required to solve the source reconstruction problem as accurately as possible. Approaches to doing so for the static case, *i.e.*, corresponding to a single time instant, are indicated, and in future work would be based on exploiting the measurements provided by the sensor network at different times and knowledge about how the locations of the sources evolve with time.

The paper is organised as follows. Section 2 gives the background, including models for the acoustic output of the sound sources and for sound propagation in the ocean, information about the sensors used for ocean sound monitoring, details of the numerical simulation experiments, and descriptions of the different approaches to estimating the acoustic noise map. In Section 3 the source reconstruction problem and how it is solved are described. Section 4 discusses the results obtained from the numerical simulation experiments. Finally, Section 5 gives conclusions and ideas for further work.

## 2. Background

### 2.1. Model for the Locations of the Sound Sources and Receivers

Although the ships and receivers could be located anywhere in the region of interest, here that region is discretised into a grid containing *M* points with the locations of the (centroids of the) ships and receivers restricted to the grid points. Suppose a shipping lane containing *N* of those grid points forms a part of the region of interest, with $n_{\mathrm{ship}}$ ships (or sources) positioned within the shipping lane and $n_{\mathrm{rcvr}}$ receivers (or sensors) located outside the lane. Let the grid points within the region of interest be indexed by $i\in I_{\mathrm{grid}}=\left\{1,\ldots ,M\right\}$, and denote their locations by the position vectors $r_{i}$. Let the grid points in the shipping lane be indexed by $j\in I_{\mathrm{lane}}\subseteq I_{\mathrm{grid}}$, where $I_{\mathrm{lane}}$ has *N* elements, and let the ships be located at grid points indexed by $k\in I_{\mathrm{ship}}\subseteq I_{\mathrm{lane}}$, where $I_{\mathrm{ship}}$ has $n_{\mathrm{ship}}$ elements. Suppose $I_{\mathrm{ship}}=I_{\mathrm{ship},k}\cup I_{\mathrm{ship},u}$, where $I_{\mathrm{ship},k}$ containing $n_{\mathrm{ship},k}$ elements identifies those ships whose locations are known (from AIS transponder information that provides GPS coordinates) and $I_{\mathrm{ship},u}$ corresponds to those ships whose locations are unknown. Finally, let the receivers be located at grid points indexed by $\ell \in I_{\mathrm{rcvr}}\subseteq I_{\mathrm{grid}}$, where $I_{\mathrm{rcvr}}$ has $n_{\mathrm{rcvr}}$ elements.

### 2.2. Models for the Acoustic Output of the Sound Sources and for Sound Propagation

The acoustic output of a continuous sound source such as a ship may be characterised in terms of its radiated sound power. For a merchant ship at modest speed, the sound power radiated into the water is typically less than 100W [21]. However, in underwater acoustics, the acoustic output of a source is historically characterised in terms of a source level, which is formulated in terms of the product of the acoustic pressure and the distance from the source, and as the level of a quantity it is expressed in decibels [22]. The source level may be related back to the sound power through knowledge of the acoustic impedance of the medium (the product of the medium density and sound speed) [21]. As a simplifying assumption, the sources are regarded as monopole point sources, where the sound power is assumed to originate from a point in the medium (for a ship, this point is located just below the water surface). Note that in acoustics, it is common to express quantities as levels using decibels (dB). The level in decibels is ten times the logarithm to base 10 of the ratio of the quantity to a reference value of that quantity, with the reference value stated. Expressed in decibels, a typical value of source level for a ship at 50 Hz might be 180 dB re 1 μPa^2^m^2^. (The units for source level used here follow the more recent convention of expressing it as a product of sound pressure and distance, which is dimensionally correct and compatible with SI units. The older and more commonly encountered convention would state this source level as “180 dB re 1 μPa at 1 m”.) The source level for a ship varies with acoustic frequency and values for a typical commercial ship may be estimated from data available in the scientific literature [20].

**Figure 1:**
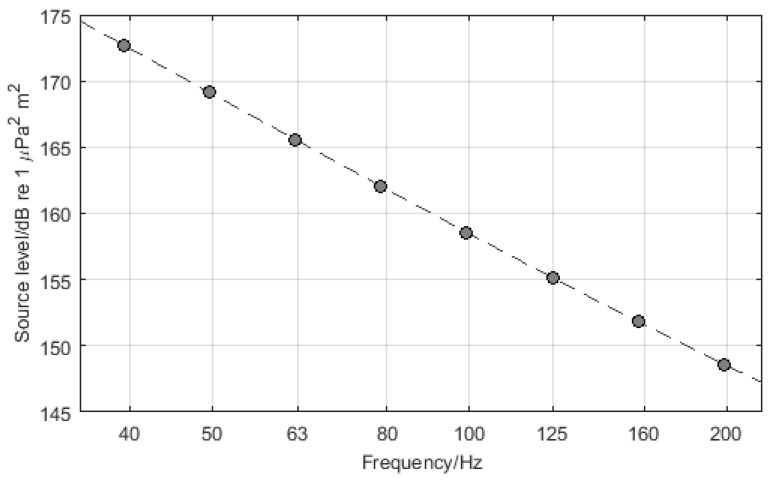
Model for the acoustic output of the sound sources evaluated at the centre frequencies of third-octave bands in the interval from 40 Hz to 200 Hz .

The study by Wales and Heitmeyer [20] provided an estimate of the average source level of an ensemble of 54 ships measured in transit. The resulting empirical source level spectrum has been used here for noise mapping purposes and is shown in Figure 1. Wales and Heitmeyer reported a dispersion of values in their measured data, with the ensemble standard deviation taking a value of about 5 dB for frequencies below 150 Hz and about 3 dB for frequencies between 400 Hz and 1200 Hz. Wales and Heitmeyer argued that their data showed negligible correlation between source level and ship speed, length or displacement. However, it is known that the source level of individual ships does depend on the speed and that the radiated noise from individual ships can show great variation depending on mechanical and geometrical design parameters [23,24,25,26]. Ships also produce tonal components from the engine and propulsion system, but these are not considered here. Instead, the source spectrum is evaluated at the centre frequencies of third-octave bands in the frequency interval from 40 Hz to 200 Hz [27], and is plotted in Figure 1. This simplification is appropriate for noise mapping for environmental impact assessment and is required in order to make the noise mapping practical [19,28]. Note that the methodology used for the numerical simulation experiments described here does not depend critically on the source model used, and a more sophisticated model may be used once a consensus is achieved on the most accurate model (this may be tractable in the future since AIS transponder data often contains information about the ship class, size and speed). For the work described here, the source level (in dB re 1 μ${\mathrm{Pa}}^{2}m^{2}$) of a ship located at grid point $r_{k},\;k\in I_{\mathrm{ship}}$, is modelled as

(1) $$S_{k}\left(f\right)=10\log_{10}\left(\frac{s_{k}}{C(f)}\right),\;C\left(f\right)=\frac{f^{3.594}}{{\left(1+{\left(f/340\right)}^{2}\right)}^{0.917}}$$

where *f* is frequency (in Hz), $s_{k}$ is a parameter representing the intercept of the source factor on the ordinate axis (in μ${\mathrm{Pa}}^{2}m^{2}$), and C(f) is a function representing the variation of the source factor with frequency. Equation (1) is a mathematical representation of the empirical data provided by Wales and Heitmeyer.

There are a number of factors that influence the propagation of sound in the ocean and contribute to the propagation loss (the reduction in signal as sound propagates from source to receiver) [29]. Broadly, these include the following: the geometrical spreading of the sound away from the source, absorption of the sound by the sea-water and the sea-bed, the interaction with the sea-surface (reflection and scattering), the interaction with (and transmission through) the sea-bed, the refraction of the sound due to the sound speed gradient, the bathymetry (water depth) between source and receiver positions, and source and receiver depth. A number of the above factors depend on the acoustic frequency, and a complex model will include frequency dependence explicitly within the model parameters. In the work described here, the propagation of sound from the ship to a position $r_{i}$ in the region of interest is described by a propagation loss function $P(f,r_{k},r_{i})$, also a function of frequency *f*, with the energy flux model of Weston [30,31] chosen for this purpose. The Weston model is particularly suitable for a shallow water channel where sound speed in the water column is assumed constant. The model calculates propagation loss in terms of energy loss using one of four simple equations that describe different propagation loss regimes according to the distance from the source and depending on the frequency and other environmental parameters in the underwater channel. The four equations are:

(2) $$\begin{array}{cc} P(f,r_{k},r_{i}) & =10\log_{10}r^{2} \end{array}$$

(3) $$\begin{array}{cc} P(f,r_{k},r_{i}) & =10\log_{10}\frac{rh_{k}h_{i}}{2h_{\min}\phi_{\min}} \end{array}$$

(4) $$\begin{array}{cc} P(f,r_{k},r_{i}) & =10\log_{10}\frac{rh_{k}h_{i}}{5.22}{\left(\alpha \int_{0}^{r}\frac{dr}{h^{3}}\right)}^{1/2} \end{array}$$

(5) $$\begin{array}{cc} P(f,r_{k},r_{i}) & =10\log_{10}\frac{rh_{k}h_{i}}{\lambda }+\frac{\lambda^{2}\alpha }{8}\int_{0}^{r}\frac{dr}{h^{3}} \end{array}$$

where *r* is the horizontal distance from the source at $r_{k}$ to the receiver at $r_{i}$, *h* is the water depth at horizontal distance *r* from the source, $h_{k}$ is the water depth at the source, $h_{i}$ is the water depth at the receiver, $h_{\min}$ is the minimum water depth between the source and receiver, $\phi_{\min}$ is the critical grazing angle, *α* is the attenuation (in dB/radian), and *λ* is the acoustic wavelength (that depends on the sound speed in water and frequency). The equations are applied to different propagation regimes moving away from the source, with Equation (2) used for spherical spreading at close range, Equation (3) for cylindrical spreading, Equation (4) for the mode stripping region, and Equation (5) for the single mode region. The environmental parameters include the bathymetry of the sea-bed, characteristics of the sea-bed such as its density and sound attenuation, the speed of sound both in water and in the sea-bed, and wind speed that governs the sea surface roughness. For this work, the following values of environmental parameters were chosen: the sound speed in water as 1490 m·s${}^{-1}$, the water depth as 30 m, the windspeed as 0 m·s${}^{-1}$, the sound speed in the sea-bed as 1816 m·s${}^{-1}$, the density of the sea-bed as 2.03 g·cm${}^{-3}$, and the attenuation in the sea-bed (assumed to be made of coarse sand) as 0.8 dB/wavelength. Note that the energy flux model is appropriate for the shallow water scenario considered here and has the benefit of range-dependence and being fast to compute. However, the methodology used for the numerical simulation experiments described does not depend critically on the propagation model used, and a different model may be used should the scenario require it (e.g., for deeper water).

It follows that the mean squared sound pressure (in $\mu {\mathrm{Pa}}^{2}$) at any position $r_{i}$ in the region of interest due to all the ships is

(6) $$s\left(f,r_{i}\right)=\sum_{k\in I_{\mathrm{ship}}}10^{\left[S_{k}\left(f\right)-P\left(f,r_{k},r_{i}\right)\right]/10}$$

In particular, the mean squared sound pressure at a receiver located at grid point $r_{\ell }$ is $s(f,r_{\ell })$, and the receiver provides a measurement of that value, which is denoted by $y(f,r_{\ell })$. Furthermore, the spatially averaged sound pressure level (in dB re $1\;\mu {\mathrm{Pa}}^{2}m^{2}$) is

(7) $$L_{p}\left(f\right)=10\log_{10}\left(\frac{1}{A_{\mathrm{ROI}}}\sum_{i\in I_{\mathrm{grid}}}s\left(f,r_{i}\right)\right)$$

where $A_{\mathrm{ROI}}$ is the area of the region of interest. For the purposes of monitoring ocean sound, the spatially averaged sound pressure level $L_{p}\left(f\right)$ defined by Equation (7) provides a measure of the total sound within the region of interest arising from the ships within the shipping lane.

### 2.3. Sensors for Ocean Sound Monitoring

An acoustic sensor used to measure ocean sound consists of several components: (i) a hydrophone, (ii) electronics that amplify and digitize the electrical signal produced by the hydrophone; and (iii) data storage systems used to record and store the data. The hydrophone is an electroacoustic transducer that produces an electrical signal in response to the sound pressure in the medium (essentially working like an underwater microphone). Typically, a hydrophone sensor element is piezoelectric in design, with a sensitivity expressed in V·Pa${}^{-1}$. Usually, the hydrophone signal requires amplification and electrical impedance buffering to drive any cable attached to the sensor without signal loss, and electronic filters are applied to the signals (a low pass filter provides an anti-aliasing function and, if needed, a high-pass filter is used to remove very low frequency artefacts in the signal such as flow noise). The signal is digitized using an analogue to digital converter with sampling rates typically being 44 kHz (although some systems offer sampling rates of hundreds of kilohertz), and with resolution typically of 16 or 24 bit. The data may be transmitted to shore via a fixed cabled link or via radio or satellite links but, more commonly, the data is stored on flash memory drives located with the hydrophone and electronics in an autonomous recorder system. The whole system requires traceable calibration, which may be achieved with an uncertainty of typically 10% (expressed as an expanded uncertainty for a coverage probability of 95%).

The key performance characteristics of the recorder system are (i) the self-noise that limits the capability of the system to measure low amplitude signals, (ii) the dynamic range that limits the ability to measure high amplitude signals faithfully; and (iii) the overall system sensitivity. For an application such as monitoring the sound from vessels in a shipping lane, it is quite feasible to choose appropriate values of the above parameters to achieve good quality data without undue distortion.

### 2.4. Numerical Simulation Experiments

For the numerical simulation experiments considered here, the region of interest is a square of size 50 km × 50 km and is discretised into a square grid containing M=501×501 points with each grid square of size 100m×100m. A shipping lane of size 50km×2km containing N=501×21 points forms a central band parallel to the sides of the region of interest. The (true) values $s_{k}$ defining the sound generated by the ships are simulated as

(8) $$s_{k}=s_{\mathrm{nom}}\left(1+\epsilon \right)$$

where $s_{\mathrm{nom}}=10^{230/10}\;\mu {\mathrm{Pa}}^{2}m^{2}$ is a nominal value for $s_{k}$, and *ϵ* is a random draw from the normal distribution with mean zero and standard deviation 30%, and the distribution is truncated to ensure $s_{k}>0$. The sea-bed is assumed to be homogenous and to be flat, which means that the propagation loss function only depends on the distance $r_{ki}$ between $r_{k}$ and $r_{i}$, *i.e.*, $P\left(f,r_{k},r_{i}\right)=P\left(f,r_{ki}\right)$. The value provided by a receiver is simulated as

(9) $$y\left(f,r_{\ell }\right)=s\left(f,r_{\ell }\right)\left(1+\delta \right)$$

where *δ* is a random draw from the normal distribution with mean zero and standard deviation $\sigma_{\mathrm{rel}}=10\;\%$, the relative standard measurement uncertainty for the receivers, and the distribution is truncated to ensure $y(f,r_{\ell })>0$. The measured values are provided at $n_{\mathrm{freq}}=8$ frequencies $f_{m}$, namely 40, 50, 63, 80, 100, 125, 160 and 200 Hz, which are the centre frequencies of third-octave bands, using $n_{\mathrm{rcvr}}=10$ receivers arranged so that there are five receivers on either side of, and oustide, the shipping lane. The value of $\sigma_{\mathrm{rel}}$ is chosen conservatively in order to investigate what might be achieved using a relatively small network of relatively poor sensors.

Four numerical simulation experiments (labelled A to D) are considered. In all experiments, there are $n_{\mathrm{ship}}=50$ ships, and the experiments differ in the number $n_{\mathrm{ship},k}$ of ships whose locations are assumed to be known and the furthest distance $d_{\max}$ of a receiver from the shipping lane as follows:

A: $n_{\mathrm{ship},k}=40$ and $d_{\max}\leq 1\;\mathrm{km}$;
B: $n_{\mathrm{ship},k}=25$ and $d_{\max}\leq 1\;\mathrm{km}$;
C: $n_{\mathrm{ship},k}=40$ and $d_{\max}\leq 24\;\mathrm{km}$;
D: $n_{\mathrm{ship},k}=25$ and $d_{\max}\leq 24\;\mathrm{km}$.

**Figure 2:**
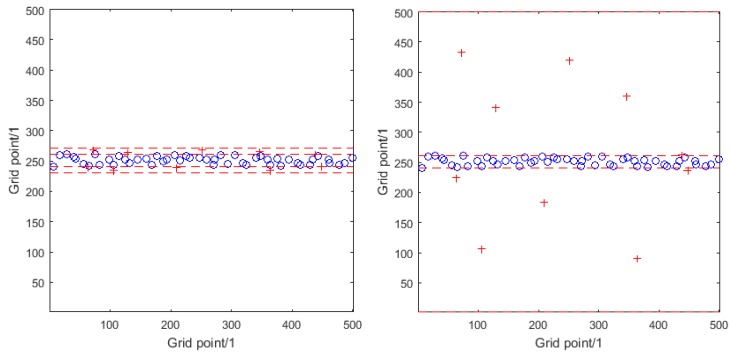
Distributions of ships (blue circles) and receivers (red crosses) within the region of interest for numerical simulation experiments A and B (**Left**) and C and D (**Right**).

Figure 2 illustrates the distributions of ships (within the central shipping lane) and receivers (outside the lane) for the different numerical simulation experiments. In each graph the central two red broken lines define the shipping lane. Additionally, there are red broken lines either side of the shipping lane that define bands in which the receivers are placed: narrow bands for numerical simulation experiments A and B and wider bands for experiments C and D. In the graph on the right the outer limits of these bands, being coincident with the top and bottom edges of the region of interest, are not too clear.

### 2.5. Approaches to Estimating Ocean Sound

#### 2.5.1. Based on Modelling

One approach to estimating the acoustic noise map makes no use of the measured values provided by the receivers. Instead it uses approximate knowledge of the parameters $s_{k}$ for those ships that are known to be in the region of interest and a model for sound propagation. For example, the parameters $s_{k}$ for the ships located at grid points $r_{k},\;k\in I_{\mathrm{ship},k}$, are all assigned to be the nominal value $s_{\mathrm{nom}}$, and an estimate $\hat{s}\left(f,r_{i}\right)$ of the mean squared sound pressure at each point $r_{i},\;i\in I_{\mathrm{grid}}$, due to those ships is calculated using Equations (1) and (6) with the summation taken over indices $k\in I_{\mathrm{ship},k}$, *i.e.*,

(10) $$\hat{s}\left(f,r_{i}\right)=\sum_{k\in I_{\mathrm{ship},k}}10^{\left[S_{k}\left(f\right)-P\left(f,r_{k},r_{i}\right)\right]/10},\;S_{k}\left(f\right)=10\log_{10}\left(\frac{s_{\mathrm{nom}}}{C(f)}\right)$$

Equation (7) is then used to calculate an estimate of the spatially averaged sound pressure level $L_{p}\left(f\right)$ in terms of the estimates $\hat{s}\left(f,r_{i}\right)$. By applying the approach a number of times to obtain different values of $L_{p}\left(f\right)$ corresponding to different values of the parameters $s_{k}$ for the ships simulated according to the statistical model in Equation (8), the influence on the estimate of $L_{p}\left(f\right)$ of uncertainty associated with knowledge about the parameters $s_{k}$ can be assessed. This assessment involves comparing the average of the values of $L_{p}\left(f\right)$ with the true value of $L_{p}\left(f\right)$ to measure the bias of the estimate, and evaluating the standard deviation of the values to measure the precision of the estimate. The approach is described as the *modelling* approach.

#### 2.5.2. Based on Measurement

Another approach makes use only of the measured values provided by the receivers. An estimate of the mean squared sound pressure at each point $r_{i},\;i\in I_{\mathrm{grid}}$, in the region of interest is given by the average of the measured values $y(f,r_{\ell })$, *i.e.*,

(11) $$\hat{s}\left(f,r_{i}\right)=\sum_{\ell =1}^{n_{\mathrm{rcvr}}}y\left(f,r_{\ell }\right)$$

Again, Equation (7) is then used to calculate an estimate of the spatially averaged sound pressure level $L_{p}\left(f\right)$ in terms of these estimates. By applying the approach a number of times to obtain different values of $L_{p}\left(f\right)$ corresponding to different measured values provided by the receivers simulated according to the statistical model in Equation (9), the influence on the estimate of $L_{p}\left(f\right)$ of uncertainty associated with the receiver measurements can be assessed. The approach is described as the *measurement* approach.

#### 2.5.3. Based on Modelling and Measurement

The approach proposed here is based on combining the knowledge about how sound propagates in the ocean with measurements of the sound made at (a small number of) discrete locations within the region of interest using a network of acoustic sensors. As described in Section 1, in a first step, the approach addresses the *source reconstruction* problem that involves providing estimates ${\hat{I}}_{\mathrm{ship}}$ and ${\hat{s}}_{k},\;k\in {\hat{I}}_{\mathrm{ship}}$ of, respectively, the locations and parameters $s_{k}$ for *all* the ships within the shipping lane (both those identified by indices in $I_{\mathrm{ship},k}$ for which there is information about their locations and parameters $s_{k}$ and those corresponding to unknown indices in $I_{\mathrm{ship},u}$ for which there is no such information). An approach to solving the source reconstruction problem is presented in Section 3. Then, in a second step, the approach addresses the *sound prediction* problem in which the solution to the source reconstruction problem is used to evaluate the mean squared sound pressure at each point $r_{i},\;i\in I_{\mathrm{grid}}$ in the region of interest, *i.e.*,

(12) $$\hat{s}\left(f,r_{i}\right)=\sum_{k\in {\hat{I}}_{\mathrm{ship}}}10^{\left[{\hat{S}}_{k}\left(f\right)-P\left(f,r_{k},r_{i}\right)\right]/10},\;{\hat{S}}_{k}\left(f\right)=10\log_{10}\left(\frac{{\hat{s}}_{k}}{C(f)}\right)$$

Again, Equation (7) is then used to calculate an estimate of the spatially averaged sound pressure level $L_{p}\left(f\right)$ in terms of these estimates. By applying the approach a number of times to obtain different values of $L_{p}\left(f\right)$ corresponding to different measured values provided by the receivers simulated according to the statistical model in Equation (9), the influence on the estimate of $L_{p}\left(f\right)$ of uncertainty associated with the receiver measurements can be assessed. The approach is described as the *modelling and measurement* approach.

## 3. Source Reconstruction

Let s denote the vector of parameters $s_{k}$ at all grid points $r_{j},\;j\in I_{\mathrm{lane}}$, within the shipping lane. Two types of information are available about the vector s. The first type is the measured values $y(f_{m},r_{\ell })$, $m=1,\ldots ,n_{\mathrm{freq}},\;\ell \in I_{\mathrm{rcvr}}$, provided by the receivers. This information is represented by the linear equations

(13) As=y

in which the matrix A, of dimension $n_{\mathrm{freq}}n_{\mathrm{rcvr}}\times N$, contains terms that depend on values of the propagation loss function $P(f_{m},r_{j\ell })$ and the function $C(f_{m})$, and the vector y contains the values $y(f_{m},r_{\ell })$. The second type is the estimate $s_{\mathrm{nom}}$ of $s_{k},\;k\in I_{\mathrm{ship},k}$, for those ships whose locations are known. This information is represented by the linear equations

(14) $$Bs=s_{\mathrm{nom}}1_{n_{\mathrm{ship},k}}$$

in which the matrix B, of dimension $n_{\mathrm{ship},k}\times N$, has a single value of unity in each row corresponding to an index of $I_{\mathrm{ship},k}$, and $1_{n_{\mathrm{ship},k}}$ is a vector of ones of dimension $n_{\mathrm{ship},k}\times 1$.

The two types of information are represented together by the linear equations

(15) Φs=z

where

(16) $$\Phi =\left[\begin{array}{c} D^{-1}A\\ E^{-1}B \end{array}\right],\;z=\left[\begin{array}{c} D^{-1}y\\ E^{-1}s_{\mathrm{nom}}1_{n_{\mathrm{ship},k}} \end{array}\right]$$

and the matrices D and E provide a weighting of the data, and are assigned to be diagonal matrices with diagonal elements equal to the standard uncertainties associated with the elements of, respectively, the vectors y and $s_{\mathrm{nom}}1_{n_{\mathrm{ship},k}}$.

Since the number $n_{\mathrm{freq}}n_{\mathrm{rcvr}}+n_{\mathrm{ship},k}$ of equations is very much smaller than the number *N* of unknowns, the Equations (15) are underdetermined, and do not possess a unique solution. (In the case of the numerical simulation experiments, $n_{\mathrm{freq}}=8$, $n_{\mathrm{rcvr}}=10$, $n_{\mathrm{ship},k}=40$ or 25, and N=501×21=10,521.) However, the knowledge that the solution is sparse, *i.e.*, that most of the elements of s are zero, can be used to provide a particular solution. This solution is defined by the vector $\tilde{s}$ that solves

(17) $$\min_{\tilde{s}}\tau ∥\tilde{s}{∥}_{1}+\frac{1}{2}{∥\tilde{\Phi }\tilde{s}-\tilde{z}∥}_{2}^{2},\;\tilde{s}\geq 0$$

where $\tilde{\Phi }$ is obtained from Φ by scaling each of its columns to have a 2-norm of one, $\tilde{z}$ is similarly obtained by scaling z to have a 2-norm of one, and s is recovered from $\tilde{s}$ using

(18) $$s_{j}={∥z∥}_{2}\frac{{\tilde{s}}_{j}}{∥\Phi_{\cdot ,j}{\left|\right|}_{2}},\;j=1,\ldots ,N$$

in which $\Phi_{\cdot ,j}$ denotes the *j*th column of Φ. In Equation (17), ${∥x∥}_{2}^{2}$ is the sum of the squares of the elements of x (the square of the vector 2-norm), and ${∥x∥}_{1}$ is the sum of the absolute values of the elements of x (the vector 1-norm). The formulation of Equation (17) in terms of $\tilde{\Phi }$ and $\tilde{z}$ corresponds to a re-parametrisation of the Equations (15) that ensures that the problem is sensibly scaled. The scalar *τ* is used to adjust the balance between satisfying the Equations (15), which represent the two types of information, and achieving sparsity of the solution, which is represented by the term $∥\tilde{s}{∥}_{1}$. The sensitivity of the solution to the choice of the value of *τ* is investigated in Section 4.

Minimisation problems of the form of Equation (17) arise in applications of compressive sensing [32], and a number of algorithms are available for solving such problems. In this work the approach and software described in [33,34] employing a homotopy-based algorithm is used. The approach allows for the inclusion of non-negativity constraints on the solution, which express the knowledge that the ships are sources (and not sinks) of sound.

However, solving Equation (17) gives estimates of the parameters $s_{k}$ for the known ships that are biased in the sense that those parameters $s_{k}$ are consistently underestimated. This property is a common feature when minimising a vector 1-norm to induce sparsity [35], as homotopy does, and the inclusion of a de-biasing step helps to improve the quality of the estimates. Let ${\hat{I}}_{\mathrm{ship}}$ contain the indices of the non-zero elements in the solution to Equation (17), which provides an estimate of the set of indices $I_{\mathrm{ship}}$ that identify the locations of the complete set of ships. The de-biasing step involves solving the non-negative linear least-squares problem

(19) $$\min_{{\tilde{s}}_{{\hat{I}}_{\mathrm{ship}}}}\frac{1}{2}∥{\tilde{\Phi }}_{{\hat{I}}_{\mathrm{ship}}}{\tilde{s}}_{{\hat{I}}_{\mathrm{ship}}}-\tilde{z}{∥}_{2}^{2},\;{\tilde{s}}_{{\hat{I}}_{\mathrm{ship}}}\geq 0$$

where ${\tilde{s}}_{{\hat{I}}_{\mathrm{ship}}}$ and ${\tilde{\Phi }}_{{\hat{I}}_{\mathrm{ship}}}$ contain, respectively, the elements of $\tilde{s}$ and the columns of $\tilde{\Phi }$ identified by the indices ${\hat{I}}_{\mathrm{ship}}$. In other words, Equation (17) involving the vector 1-norm is used only to obtain the index set ${\hat{I}}_{\mathrm{ship}}$ that estimates the locations of the ships, and Equation (19) involving only the vector 2-norm is then solved to obtain estimates of the parameters $s_{k}$ for those ships. The impact of the be-biasing step is illustrated in Section 4.

## 4. Results and Discussion

### 4.1. Source Reconstruction

**Figure 3:**
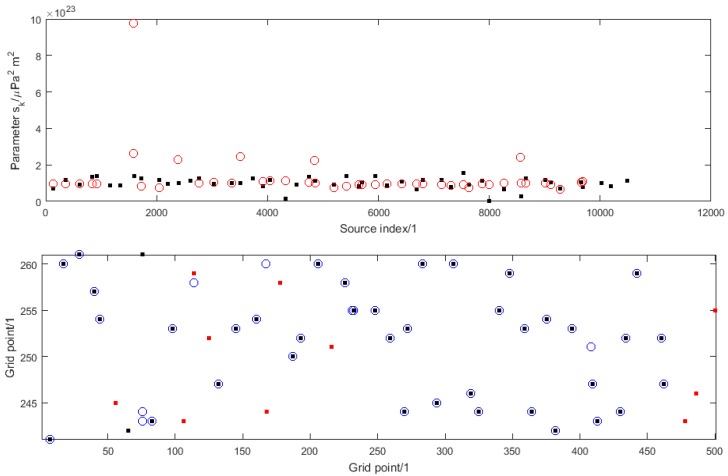
For numerical simulation experiment A, the solution to the source reconstruction problem: (**Top**) the true values (black dots) and estimated values (red circles) of the parameters $s_{k}$; (**Bottom**) the true locations within the shipping lane of the known ships (black dots) and unknown ships (red dots) and the estimated locations of the ships (blue circles).

Figure 3 illustrates the solution to the source reconstruction problem for one instance of numerical simulation experiment A and how it compares with the true solution used as the basis of the simulation. The top graph shows the true values (as black dots) and estimated values (as red circles) of the parameters $s_{k}$ plotted against, respectively, the index sets $I_{\mathrm{ship}}$ and ${\hat{I}}_{\mathrm{ship}}$. The bottom graph shows the true locations of the known ships within the shipping lane (as black dots), the true locations of the unknown ships (as red dots) and the estimated locations of all the ships (as blue circles). (Note the different scales on the horizontal and vertical axes, since in reality the shipping lane is long and thin with an aspect ratio of 50 km to 2 km, which exagerates distances in the vertical direction compared with distances in the horizontal direction.) In this case the estimated number of ships is 44.

The source reconstruction problem is not solved particularly well. Most estimates of the parameters $s_{k}$ have values close to the nominal value $s_{\mathrm{nom}}$ with a few taking quite large values. Whereas most of the known ships are correctly located (where a black dot coincides with a blue circle in the bottom graph), not one of the unknown ships is correctly located. In fact, the reconstruction encourages the estimation of a group of unknown ships close to each other by a single ship with a large value for its parameter $s_{k}$. Note that the information about the locations and parameters $s_{k}$ for the known ships is incorporated in the form of a set of observations that strongly encourages sources at the known locations. However, the known locations are not imposed as hard constraints, and so there is no guarantee that all the known locations will be identified correctly.

Indeed, the fact that the source reconstruction problem is not solved particularly well is unsurprising. It can be shown [36] that if $\tilde{\Phi }$ is a matrix of dimension n×N of normally-distributed values, recovery of a solution comprising *κ* non-zero components would require

(20) $$n>2\kappa \log\left(\frac{N}{n}\right)$$

In this ideal case, and taking $n=n_{\mathrm{freq}}n_{\mathrm{rcvr}}+n_{\mathrm{ship},k}=120$ and N=10,521, recovery is possible only for κ<14, which is much smaller even than the number of known ships. Moreover, for a matrix $\tilde{\Phi }$ of this particular form, the correlation associated with the estimates of any two components of $\tilde{s}$ is expected to be weak. In terms of the source reconstruction problem, such a situation would correspond to being able to distinguish the locations of the ships very well. However, as mentioned above, groups of unknown ships close to each other are estimated by a single ship (with a large value for its parameter $s_{k}$), which suggests some of the correlations are strong.

To examine the strength of the correlation between pairs of estimates of the parameters $s_{k}$ corresponding to different locations, define the coherence matrix C by

(21) $$C={\tilde{\Phi }}^{⊤}\tilde{\Phi }$$

**Figure 4:**
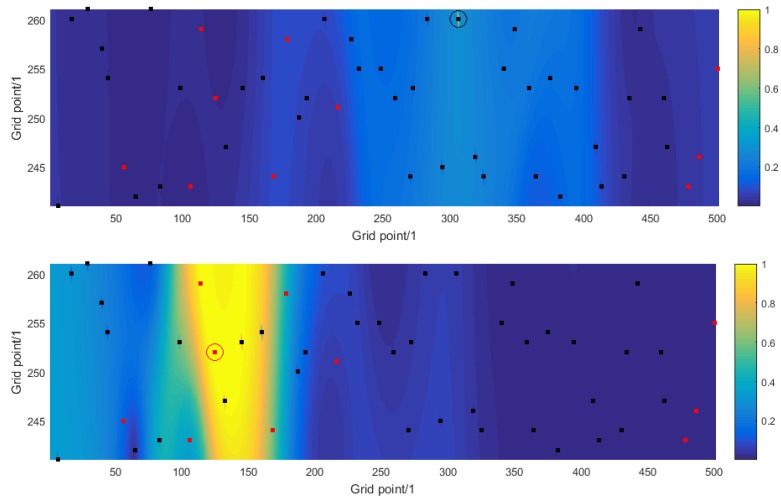
For numerical simulation experiment A, the solution to the source reconstruction problem: (**Top**) the coherence of a known ship, and (**Bottom**) the coherence of an unknown ship, with all other possible sound sources within the shipping lane.

The *i*th row (or column) of C describes the coherence (or correlation) of a sound source at a location in the shipping lane specified by the index *i* with sound sources at every other location in the shipping lane. Figure 4 shows the coherence information as contour plots for two choices of index *i*. In the top graph the index *i* corresponds to a known ship whose location has been identified correctly, and the coherence decays rapidly from the location of the ship (shown by the black dot and enclosing circle). In contrast, in the bottom graph the index *i* corresponds to an unknown ship whose location has not been identified, and the coherence decays much more slowly from the location of the ship (shown by the red dot and enclosing circle) and not at all across the shipping lane. There are a number of sound sources that are highly correlated with the ship and that, consequently, are difficult to recover separately.

**Figure 5:**
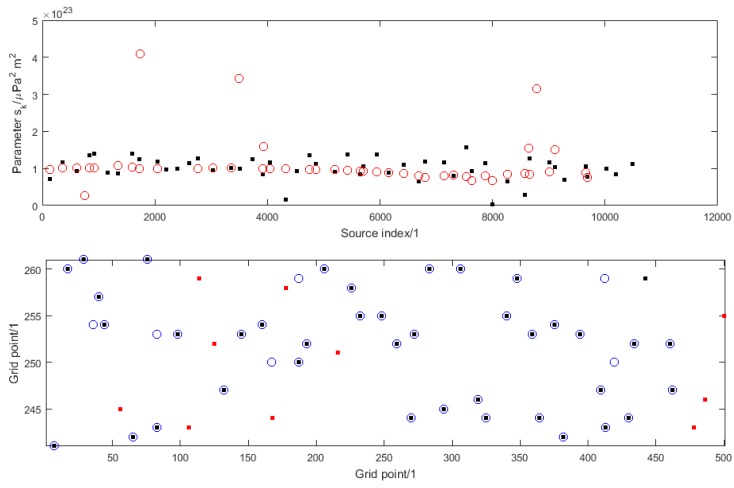
For numerical simulation experiment C, the solution to the source reconstruction problem: (**Top**) the true values (black dots) and estimated values (red circles) of the parameters $s_{k}$; (**Bottom**) the true locations within the shipping lane of the known ships (black dots) and unknown ships (red dots) and the estimated locations of the ships (blue circles).

**Figure 6:**
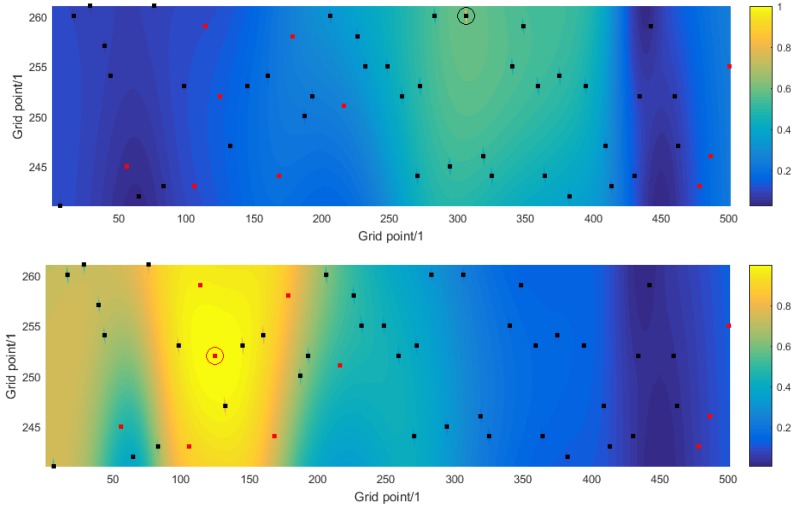
For numerical simulation experiment C, the solution to the source reconstruction problem: (**Top**) the coherence of a known ship, and (**Bottom**) the coherence of an unknown ship, with all other possible sound sources within the shipping lane.

Figure 5 and Figure 6 illustrate the same information as in Figure 3 and Figure 4 but for one instance of numerical simulation experiment C where the receivers are more widely spread and located further away from the shipping lane than for experiment A. The results are qualitatively very similar, except that the coherence functions decay somewhat slower. It follows that there is a spatial resolution to which a sound source can be identified. Moreover, that spatial resolution is itself spatially dependent (because it is different for the two choices of index *i*) and it depends on the information about the sound sources (how many ships have known locations) and on the design of the network of receivers.

### 4.2. Sound Prediction

**Figure 7:**
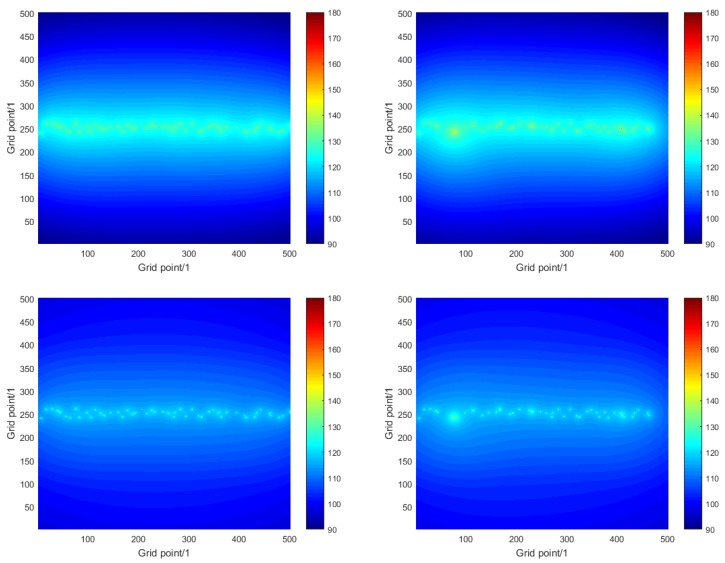
For numerical simulation experiment A, the true acoustic noise map for frequencies 63 Hz (**Top-Left**) and 125 Hz (**Bottom-Left**), and the corresponding estimates of the noise map (**Top-Right** and **Bottom-Right**) obtained using the analysis approach based on modelling and measurement.

**Figure 8:**
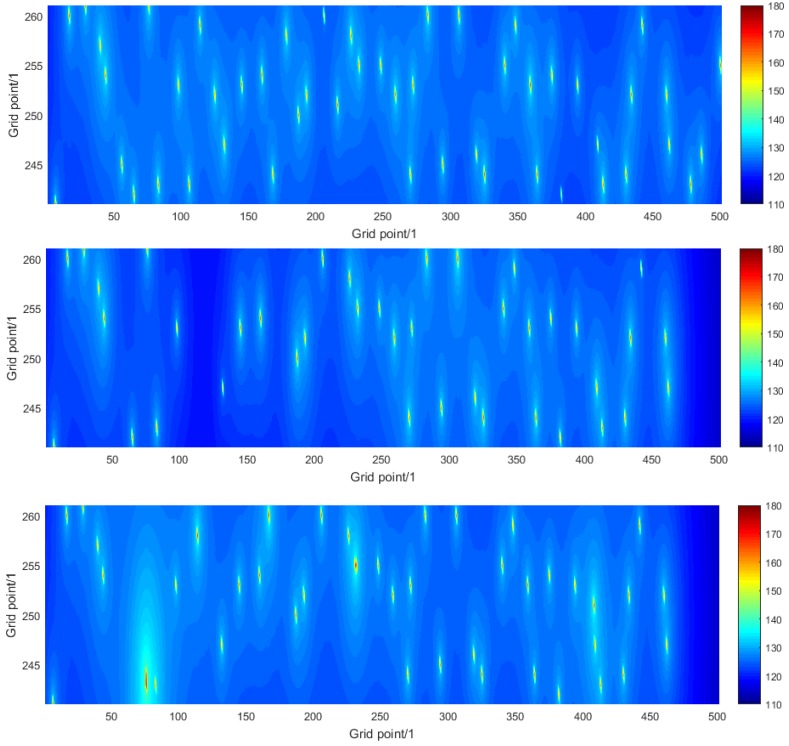
For numerical simulation experiment A, detail of the acoustic noise maps within the shipping lane for the frequency 63 Hz: true noise map (**Top**), and corresponding estimates obtained using the analysis approach based on modelling (**Middle**) and modelling and measurement (**Bottom**).

Figure 7 illustrates the solution to the sound prediction problem for one instance of numerical simulation experiment A and how it compares with the true solution used as the basis of the simulaton. The graphs on the left show the true acoustic noise maps for the region of interest and the frequencies of 63 Hz and 125 Hz. The graphs on the right show the corresponding estimates of the noise maps obtained using the modelling and measurement approach. The sound sources are clearly evident, and also that their influence is largely restricted to the shipping lane, which is a result of the nature of the propagation loss function that decays rapidly with distance. The differences between the top and bottom graphs illustrate the frequency dependence of both the source levels of the ships and the sound propagation. Figure 8 illustrates the detail of the acoustic noise maps within the shipping lane for the single frequency of 63 Hz. The graphs correspond to the true noise map (top) and the estimates obtained on the basis of the modelling approach (middle) and modelling and measurement approach (bottom). Results for the measurement approach are not shown because the noise map is constant over the region of interest in this case. (In these graphs the ships do not appear as circles, which is a result of the difference in scales in the horizontal and vertical directions.) It is seen in the middle graph that there are parts of the region of interest, e.g., the vertical strip between grid points 100 and 200 in the horizontal direction, where the values of the acoustic noise map are underestimated, which correspond to ships that are missing because they are unknown. It is seen in the bottom graph that the modelling and measurement approach has gone some way to improve the estimation in those parts by using the measured data provided by the sensor networks to reconstruct those unknown ships. However, in other parts of the region of interest, the acoustic noise map is overestimated, where a single reconstructed ship has a large source level.

Table 1 summarises the estimates of spatially averaged sound pressure level $L_{p}\left(f\right)$ provided by the analysis approaches based on modelling, measurement and a combination of modelling and measurement for two of the considered frequencies. For each numerical simulation experiment, the approaches are applied to one hundred instances of simulated data, and the average $\mu [L_{p}\left(f\right)]$ and standard deviation $\sigma [L_{p}\left(f\right)]$ of the values obtained are calculated. In the table are given the true value $L_{p}^{*}\left(f\right)$, the difference $\delta =\mu \left[L_{p}\left(f\right)\right]-L_{p}^{*}\left(f\right)$ and the standard deviation $\sigma =\sigma [L_{p}\left(f\right)]$.

The results presented in the table indicate that the estimate of $L_{p}\left(f\right)$ provided by the modelling approach is adversely affected by reducing the number $n_{\mathrm{ship},k}$ of ships whose locations are known (compare the results for numerical simulation experiments A and C with those for experiments B and D), whereas the estimate provided by the measurement approach is adversely affected by moving the receivers away from the shipping lane (compare the results for numerical simulation experiments A and B with those for experiments C and D). In contrast, the approach based on both modelling and measurement gives results that are in comparison insensitive to the value of $n_{\mathrm{ship},k}$ and to the locations of the receivers. Furthermore, the combination of modelling and measurement gives estimates that are better than those provided by the other approaches, in terms of their closeness of agreement with the true spatially averaged sound pressure level and the dispersions of results. Only in the case of numerical simulation experiment A does the approach based on both modelling and measurement give results for which the difference between average and true values is large compared to the standard deviation of the values.

### 4.3. Sensitivity to the Choice of *τ*

Recall that the parameter *τ* in Equation (17) controls the balance between satisfying Equations (15) and achieving sparsity of the solution. The default value recommended for the software used to solve Equation (17) [33] is

(22) $$\tau =\rho^{2}\sqrt{\log N}$$

where *ρ* is the standard deviation of the errors in the observed data comprising $\tilde{z}$. Since the evaluation of $\tilde{z}$ involves a weighting of the observed data (by the inverse standard uncertainties associated the data) and a scaling (by the 2-norm of z), the default value is

(23) $$\tau =\frac{\sqrt{\log N}}{{∥z∥}_{2}}$$

**Figure 9:**
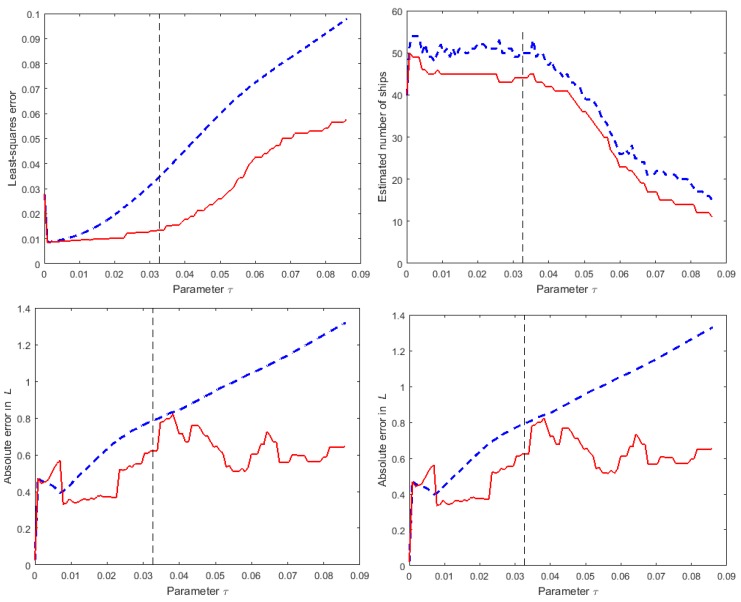
For numerical simulation experiment A, the sensitivity of results to the choice of *τ*: (**Top-Left**) value of the least-squares error $∥\tilde{\Phi }\tilde{s}-\tilde{z}{∥}_{2}^{2}$; (**Top-Right**) estimated number of ships; (**Bottom-Left**) absolute error (in dB) for the predicted values of T(f=63Hz) and (**Bottom-Right**) for T(f=125Hz). The blue broken lines and red solid lines show results for the solutions, respectively, before and after the de-biasing step.

Figure 9 shows the influence of the choice of the parameter *τ* on the results obtained for one instance of numerical simulation experiment A and compares the results without and with the de-biasing step. The graphs show the dependence on *τ* of the least-squares error $∥\tilde{\Phi }\tilde{s}-\tilde{z}{∥}_{2}^{2}$, the estimated number of ships, and the absolute errors in the predicted values of spatially averaged sound pressure level for two of the considered frequencies.

As *τ* is decreased more emphasis is given to solving (weighted versions of) Equations (13) and (14) rather than to the sparsity of the solution, so that the least-squares error decreases but the estimated number of ships increases. Note that the estimated number of ships after the de-biasing step is smaller than the estimated number before the step because the non-negativity constraints in Equation (19) increases the sparsity of the solution. As *τ* is increased more emphasis is given to the sparsity of the solution and when the estimated number of ships falls below $n_{\mathrm{ship},k}$ the least-squares error rapidly increases because Equation (14) containing information about the known ships cannot be satisfied.

**Figure 10:**
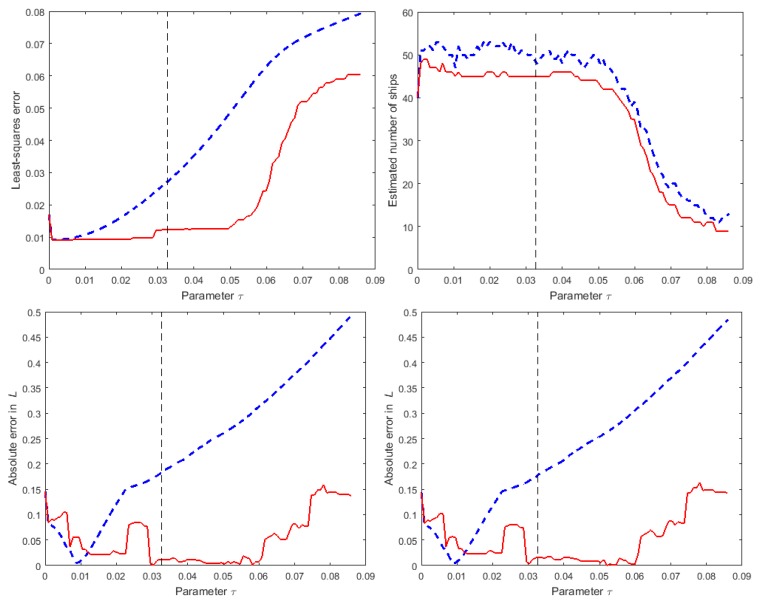
For numerical simulation experiment C, the sensitivity of results to the choice of *τ*: (**Top-Left**) value of the least-squares error $∥\tilde{\Phi }\tilde{s}-\tilde{z}{∥}_{2}^{2}$; (**Top-Right**) estimated number of ships; (**Bottom-Left**) absolute error (in dB) for the predicted values of T(f=63Hz) and (**Bottom-Right**) for T(f=125Hz). The blue broken lines and red solid lines show results for the solutions, respectively, before and after the de-biasing step.

Figure 10 illustrates the same information as in Figure 9 but for one instance of numerical simulation experiment C. The results are qualitatively very similar, but the improvement in the estimates of $L_{p}\left(f\right)$ obtained from the de-biasing step are very evident. The value used for *τ* to generate the results in Section 4.1 and Section 4.2 is marked on the graphs as a vertical broken line. In both figures, the value is in an interval of values for *τ* within which the results are generally insensitive to the choice of *τ*. Consequently, for the two strategies for locating the receivers employed in the numerical simulation experiments, the recommended value for *τ* would appear to be appropriate.

## 5. Conclusions and Future Work

An approach has been described that uses measurements of ocean sound provided by a network of acoustic sensors, partial information about the locations and sound levels of the sources of the sound, and models of the acoustic output of the sources and for sound propagation in the ocean, to estimate the sound field produced by the sources anywhere within a region of interest. Aggregated measures of the sound field, such as the spatially averaged sound pressure level, can then be calculated. The approach involves a source reconstruction step, which uses a compressive sensing algorithm to exploit the sparsity of the representation of the sound field in terms of the sources, and a sound prediction step. A set of numerical simulation experiments has been used to compare the results obtained using the approach with those provided by other approaches, such as based on modelling alone and measurement alone, as well as to investigate the influence of the design of the sensor network on the results. Although the spatial resolution to which the source reconstruction problem can be solved is generally limited, reliable estimates of aggregated measures of the sound field can be obtained compared to those provided by the other approaches.

The focus has been on the *static* problem, *i.e.*, a problem relating to a single instant in time. In practice, measurements of ocean sound are available from the sensor network for a sequence of time instants, and there is also partial information about how the locations and sound levels of the sources evolve in time, for example, from the AIS and assuming the sound levels remain constant. There is interest in how aggregated measures of the sound field change with time to know whether there are temporal trends in those measures. Moreover, the increase in the volume of available data can be expected to improve the accuracy to which the source reconstruction can be solved (which becomes a source tracking problem in the *dynamic* case) and, consequently, the estimate of the sound field (and its evolution in time). Future work will consider this dynamic problem, and there are various possibilities to treat it including, for example, the overlapping group lasso algorithm [37] or by combining the compressive sensing algorithm described with a Kalman filter algorithm.

Of course, sources other than ships, such as airgun sources used for geophysical surveying and marine pile driving producing construction noise, will radiate in the frequency interval considered. Some of these sources are intermittent, but since the noise mapping process is essentially one of adding incoherent sound energy contributions, the contributions of these sources can be modelled separately and added to form the overall noise map. The work described here was restricted to one source (viz., ship traffic), but it may be extended to other sources assuming that a suitable model for those sources exists.

## Figures and Tables

**Table 1:** For the four numerical simulation experiments and two frequencies *f* (in Hz), estimates (in dB re $1\;\mu {\mathrm{Pa}}^{2}m^{2}$) of the spatially averaged sound pressure level $L_{p}\left(f\right)$ obtained using the analysis approaches based on modelling (Mod.), measurement (Meas.) and modelling and measurement (compressive sensing, CS). Listed are the true value $L_{p}^{*}\left(f\right)$, the difference $\delta =\mu \left[L_{p}\left(f\right)\right]-L_{p}^{*}\left(f\right)$ and the standard deviation $\sigma =\sigma [L_{p}\left(f\right)]$, where $\mu [L_{p}\left(f\right)]$ and $\sigma [L_{p}\left(f\right)]$ are the average and standard deviation, respectively, of the values obtained by applying the approaches to one hundered instances of simulated data.

			A		B		C		D	
			$n_{\mathrm{ship},k}=40$		$n_{\mathrm{ship},k}=25$		$n_{\mathrm{ship},k}=40$		$n_{\mathrm{ship},k}=25$	
			$d_{\max}\leq 1\;\mathrm{km}$		$d_{\max}\leq 1\;\mathrm{km}$		$d_{\max}\leq 24\;\mathrm{km}$		$d_{\max}\leq 24\;\mathrm{km}$	
*f*	$L_{p}^{*}\left(f\right)$		δ	σ	δ	σ	δ	σ	δ	σ
63	182.73	Mod.	−1.01	0.20	−3.03	0.24	−1.01	0.20	−3.03	0.24
		Meas.	−3.73	0.16	−3.74	0.17	−10.51	0.23	−10.55	0.24
		CS	0.41	0.13	0.25	0.16	−0.03	0.09	−0.04	0.08
125	172.68	Mod.	−1.00	0.20	−3.02	0.24	−1.00	0.20	−3.02	0.24
		Meas.	−2.12	0.17	−2.12	0.16	−7.97	0.21	−7.98	0.22
		CS	0.41	0.13	0.25	0.16	−0.03	0.09	−0.03	0.08
